# Production of Bioactive Peptides from Tartary Buckwheat by Solid-State Fermentation with *Lactiplantibacillus plantarum* ATCC 14917

**DOI:** 10.3390/foods13193204

**Published:** 2024-10-09

**Authors:** Panpan Wang, Tingjun Ma

**Affiliations:** College of Food Science and Engineering, Beijing University of Agriculture, Beijing 102206, China; wangpanpan12369@163.com

**Keywords:** buckwheat peptide, solid-state fermentation, response surface methodology

## Abstract

Buckwheat is a valuable crop that contains various nutrients and functional components. Tartary buckwheat peptide is a protease-hydrolyzed protein with a wide range of physiological functions. Tartary buckwheat peptide produced through microbial fermentation can decrease the enzymatic digestion of buckwheat protein, which contributes to the bitter taste, and improve both the flavor and texture of buckwheat peptide products. In this study, microbial fermentation using probiotics was employed to prepare Tartary buckwheat peptides, and the preparation process was optimized. Based on single-factor experiments, the polypeptide content in the fermentation solution initially increased and then decreased with varying water content, inoculum concentration, glucose addition, fermentation temperature, fermentation time, and potassium dihydrogen phosphate addition. According to the response surface methodology, the maximum peptide content was achieved under fermentation conditions of 60.0% moisture content, 12.87% inoculum ratio, 2.0% glucose, and a fermentation temperature of 30.0 °C, with an actual value of (22.18 ± 1.02) mg/mL. The results show that fermentation with *Lactiplantibacillus plantarum* produces higher peptide levels and is safer than other microbial fermentation methods.

## 1. Introduction

Buckwheat(*Fagopyrum tataricum* Gaertn.) belongs to the Polygonaceae family and includes annual and perennial dicotyledonous plants. Tartary buckwheat is rich in nutrients and provides therapeutic benefits [1]. With a protein concentration ranging from 11.8% to 15.83%, Tartary buckwheat surpasses other food crops such as corn, rice, and sorghum [2]. Eighteen amino acids have been identified in Tartary buckwheat, with notably high levels of lysine and eight essential amino acids [3]. However, the current utilization of Tartary buckwheat is limited. A portion of the buckwheat is used to extract flavonoids, phenolic acids, and other medicinal compounds, while the protein-containing portion of buckwheat pomace is often discarded. Another part of Tartary buckwheat is processed into buckwheat products, but the presence of trypsin inhibitors, saponins, tannins, and other anti-nutritional factors reduces the digestibility of buckwheat protein. This significantly impacts the consumption of buckwheat products, leading to the underutilization of resources [4]. Although enzymatic digestion of proteins can address these issues, enzymes are expensive, and the resulting products often contain bitter peptides. Using the residue left after extracting flavonoids and phenolic acids from Tartary buckwheat as the raw material and applying microbial fermentation to produce bioactive buckwheat peptides can help resolve this problem. This approach not only reduces resource waste but also reduces the bitter taste associated with enzymatic digestion of buckwheat protein. It effectively addresses the challenges of low buckwheat protein digestibility, meeting the growing demand for functional foods for infants, young children, middle-aged individuals, and the elderly.

Bioactive peptides are functional substances composed of covalently bonded amino acid residues, typically forming high-molecular-weight compounds [5,6]. Many peptides can influence microbial activity and increase the body’s metabolism. They possess various bioactivities, including antioxidant [7], antimicrobial [8], anti-obesity, anti-hypertensive [9], antitumor [10], anti-inflammatory [11], and anti-diabetic properties [12,13]. Bioactive peptides derived from food proteins show great potential for use as functional food ingredients or nutraceuticals [14]. Additionally, buckwheat protein exhibits superior functional properties, such as a higher nitrogen solubility index, and better water-holding, emulsifying, and foaming capacities compared to soy protein isolate [15,16].

The preparation methods for bioactive peptides primarily include enzymatic hydrolysis [17], microbial fermentation [18], chemical extraction [19], and artificial synthesis [20]. Among these, enzymatic hydrolysis and microbial fermentation are the two methods most commonly used in industrial production. However, enzymatic hydrolysis tends to produce hydrophobic groups, resulting in a bitter taste, which greatly limits the application of bioactive peptides in the food industry. Microbial fermentation, on the other hand, offers advantages by producing a variety of complex flavor components, such as amino acids, vitamins, and organic acids, which together improve the flavor, taste, and other properties of the fermentation mash. Despite these benefits, microbial fermentation also has its drawbacks. The primary concern is safety, as this limits the preparation of bioactive peptides via microbial fermentation. The number of non-toxic, food-grade bacteria is relatively small, and it remains unclear whether many strains with strong protease production capabilities are safe for human consumption. Buckwheat provides a good substrate for the growth and survival of probiotics [21], which is why probiotics are used in the preparation of Tartary buckwheat bioactive peptides.

The aim of this study was to select suitable probiotics for the preparation of Tartary buckwheat peptides and to explore the effect of different fermentation conditions on the peptide content of Tartary buckwheat.

## 2. Materials and Methods

### 2.1. Research Materials

*Lactiplantibacillus plantarum* ATCC 14917, *Levilactobacillus brevis* CGMCC 1.214, and *Lactococcus lactis* CGMCC 1.62 were purchased from the China General Microbiological Culture Collection Center (Beijing, China), CGMCC. Tetrapeptide standard (Gly-Gly-Tyr-Arg) and trichloroacetic acid (TCA) were obtained from Sigma-Aldrich (St. Louis, MO, USA). Copper sulfate (CuSO_4_ 5H_2_O), sodium tartarate (KNaC_4_H_4_O_6_ 4H_2_O), and glucose were supplied by Solarbio Science & Technology (Beijing, China). Sodium chloride (NaCl) was purchased from the Beijing Chemical Plant (Beijing, China).

### 2.2. Buckwheat Crude Protein Sample Preparation

Buckwheat proteins were primarily isolated using a solubilization—precipitation method. Buckwheat grains were milled into flour. A 50 g portion of the flour was added to 1 L of distilled water and stirred at 50 rpm. The solution was adjusted to pH 8 with NaOH. Alkaline protease (40.6 U/g) was then added, and the mixture was extracted at 46 °C for 20 min to dissolve the protein. The dispersion was stirred at 140 rpm for 2 h to extract the proteins, followed by centrifugation at 9000 rpm for 12 min. The precipitate was discarded, and the supernatant was collected. The pH of the supernatant was adjusted to 4.5 using 20% hydrochloric acid. The resulting pellet was washed twice with distilled water. The protein pellet was then resuspended in distilled water and neutralized to pH 7.0 with NaOH. The pellet was dialyzed using a dialysis membrane with a molecular weight cutoff of 8000–14,000 Da. The samples were freeze-dried and stored at 4 °C, yielding the crude extract of Tartary buckwheat protein.

### 2.3. Fermentation Preparation

The De Man, Rogosa, and Sharpe (MRS) formula per liter consists of: 10.0 g peptone, 8.0 g beef extract, 4.0 g yeast extract, 20.0 g glucose, 2.0 g dipotassium hydrogen phosphate, 2.0 g diammonium hydrogen citrate, 5.0 g sodium acetate, 0.2 g magnesium sulfate, 0.04 g manganese sulfate, and 1.0 mL Tween 80. The pH was adjusted to 6.2 ± 0.2.

All beakers, shake flasks, centrifuge tubes, pipette tips, and other apparatus were sterilized at 121 °C for 20 min before use. Strains (0.1 g) were incubated in 50 mL of liquid MR medium at 30 °C for 18 h. After fermentation, the medium was centrifuged at 1510 rpm for 10 min, and the pellet was washed twice with 0.85% physiological saline before being resuspended in 50 mL of sterile distilled water.

The cell viability of the strain suspensions was determined by spreading the above dilutions on MRS agar plates. Based on the cell viability results, the strain suspensions were diluted to a concentration of 2 × 10^7^ CFU/mL.

### 2.4. Peptide Fermentation Process

Solid-state fermentation (SSF) was conducted in 500 mL conical flasks containing 2 g of Tartary buckwheat crude protein medium. The strains, inoculated at 10~15% (*v*/*v*), were introduced into the medium. The fermentation ratio for mixed strains was 1:1. The incubator temperature was set to 28~30 °C. The conical flasks were sealed with a sealing film and placed in the incubator for 48 h of continuous fermentation.

### 2.5. Determination of Peptide Content in Fermentation Supernatant

The biuret method was used to determine the content of buckwheat peptides in the fermentation supernatant. To prepare the bisulfite reagent, 1.5 g CuSO_4_-5H_2_O and 6 g KNaC_4_H_4_O_6_-4H_2_O were dissolved in 500 mL of double distilled water (DDW), followed by the addition of 300 mL of 10% NaOH solution, and the mixture was thoroughly mixed. A peptide standard solution (10 mg/mL) was used, with 0.0 mL, 0.2 mL, 0.4 mL, 0.6 mL, 0.8 mL, and 1.0 mL taken into clean test tubes, each replenished to 1 mL with DDW. Then, 4 mL of bisulfite reagent was added. The mixture was shaken thoroughly, left to stand at room temperature for 30 min, and the absorbance was measured at 540 nm. The R^2^ value of the standard curve was 0.9994.

(1) Y=0.05615x−0.0011

For the fermentation supernatant, an equal volume of 10% TCA was added and left at room temperature for 20 min. The mixture was then centrifuged at 5000 rpm for 20 min. From the resulting supernatant, 1 mL was taken in a stoppered test tube, and 4 mL of bicuculline reagent was added. The mixture was shaken well and left to stand at room temperature for 30 min, after which the peptide content in the fermentation mash was determined.

### 2.6. Fermentation Preparation of Buckwheat Peptides Single-Factor Experiments

Single-factor experiments were conducted to analyze the effects of glucose addition, inoculum ratio, potassium phosphate addition, moisture content, fermentation temperature, and fermentation time on peptide extraction yield. Following the method described in Section 2.4, glucose was added at concentrations of 0.5%, 1.0%, 1.5%, 2.0%, 2.5%, and 3.0%. Inoculum ratios were varied at 10%, 11%, 12%, 13%, 14%, and 15%. Potassium phosphate was added at 0.1%, 0.2%, 0.3%, 0.4%, 0.5%, and 0.6%. Moisture content was adjusted to 30%, 40%, 50%, 60%, 70%, and 80%. Fermentation temperatures were tested at 24 °C, 26 °C, 28 °C, 30 °C, 32 °C, 34 °C, 36 °C, and 38 °C, and fermentation times were set at 1, 2, 3, 4, 5, 6, and 7 days. The yield of buckwheat peptides was studied under these fermentation conditions using the control variable method to identify the optimal single-factor conditions for peptide extraction. While one factor was varied, the others were kept constant, specifically with *Lactiplantibacillus plantarum*, 2.0% glucose addition, 13% inoculum, 0.2% potassium dihydrogen phosphate, 60% moisture content, a fermentation temperature of 30 °C, and a fermentation time of 48 h.

### 2.7. Buckwheat Peptide Response Surface Optimization Test

Based on the results of the single-factor experiments and significance analysis between the factors, the main factors affecting the preparation of buckwheat peptides via fermentation were identified as moisture content (*A*), glucose addition (*B*), inoculum ratio (*C*), and fermentation temperature (*D*). A four-factor response surface test was conducted with peptide yield as the key indicator. The factors and levels are shown in Table 1.

### 2.8. Statistical Analysis

All results are expressed as mean ± the standard deviation (SD) plotted using Origin 2019b. The experiment was repeated three times and the data was analyzed once. Analysis of variance (ANOVA) and Duncan’s multiple comparison tests were used to determine significant differences among means (*p* < 0.05) using IBM SPSS 20.0 Statistics.

## 3. Results

### 3.1. Strain Selection

**Figure 1:**
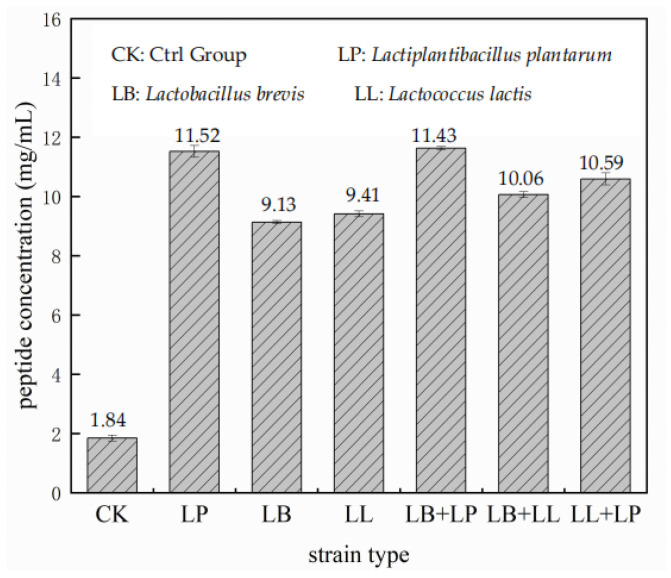
Effect of different strains on peptide content.

The peptide content in the control group after 48 h of fermentation is shown in Figure 1. *Lactiplantibacillus plantarum* produced the highest peptide level at 11.52 mg/mL, followed closely by the mixed strain of *Lactiplantibacillus plantarum* and *Levilactobacillus brevis* at 11.43 mg/mL. After 48 h of fermentation, the ability of a single *Lactobacillus* strain to produce peptides was ranked as *Lactiplantibacillus plantarum* > *Lactococcus lactis* > *Levilactobacillus brevis*. Therefore, *Lactiplantibacillus plantarum* was determined to be the most suitable bacterium for peptide synthesis.

### 3.2. Results of the Univariate Experiment

#### 3.2.1. Glucose Addition’s Effect on Peptide Concentration in Fermentation Mash

**Figure 2:**
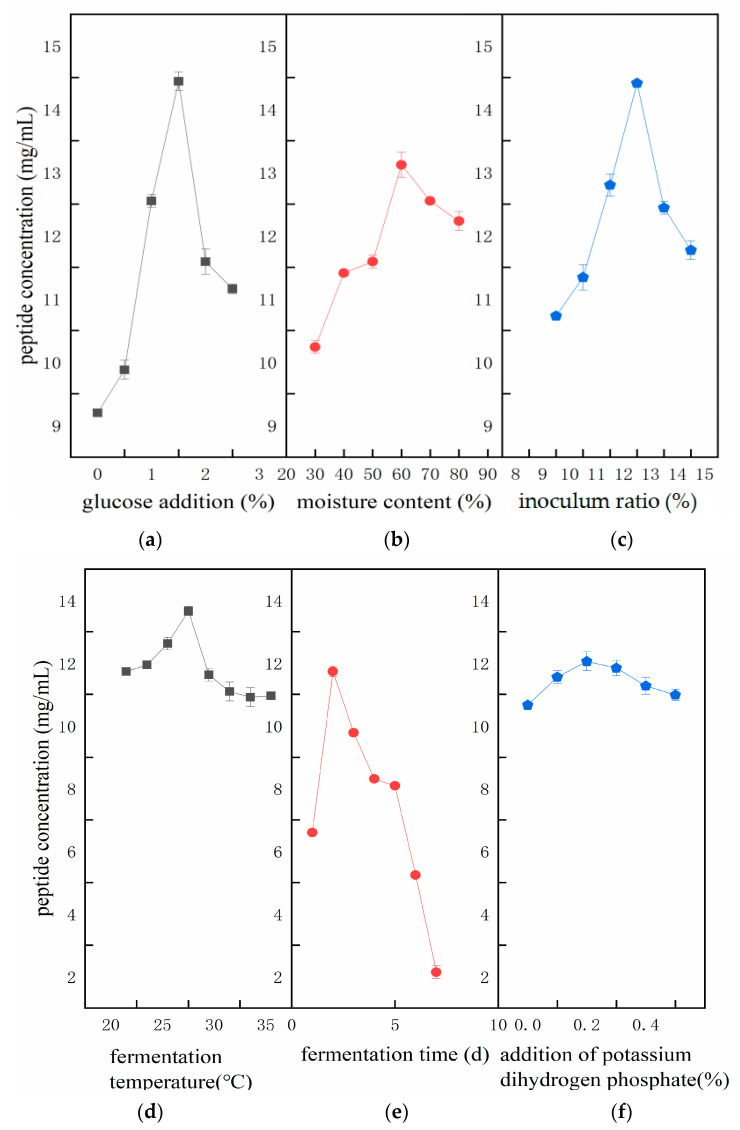
Effect of different fermentation conditions on peptide content in fermentation broth.

Figure 2a shows that the buckwheat peptide content in the fermentation mash gradually increased as the glucose addition increased from 0.5% to 2.0%. The highest peptide content, (14.8 ± 0.35) mg/mL, was achieved at 2.0% glucose addition, which was significantly higher than at other levels (*p* < 0.05). However, when glucose addition exceeded 2.0%, the peptide content began to decline. Glucose acts as a carbon source in the fermentation medium, supporting the growth of *Lactiplantibacillus plantarum* by promoting extracellular protease formation, which promotes peptide production. However, excessive glucose may accelerate the respiration rate of microorganisms, lowering the pH of the culture environment and ultimately inhibiting the growth of *Lactiplantibacillus plantarum* and enzyme synthesis. Therefore, 2.0% was selected as the optimal glucose addition amount.

#### 3.2.2. Moisture Content’s Effect on Peptide Content in Fermentation Mash

As shown in Figure 2b, the peptide content steadily increased as the moisture content rose from 30% to 60%. The highest peptide content, (13.62 ± 0.23) mg/mL, was achieved at 60% moisture content. However, as moisture content increased from 60% to 80%, peptide content significantly decreased. In a semi-liquid and semi-solid medium, moisture concentration is crucial for microbial growth. When the moisture content is too low, bacterial growth is restricted, limiting the production of extracellular enzymes and resulting in uneven and unstable fermentation. Therefore, 60% moisture content was selected for subsequent studies.

#### 3.2.3. Inoculum Ratio’s Effect on Peptide Content in Fermentation Mash

As shown in Figure 2c, peptide content in the fermentation mash increased as the inoculum ratio rose from 10% to 13%, after which it began to decline. This may be because, with inoculum ratio below 13%, the *Lactiplantibacillus plantarum* population is insufficient to fully ferment the substrate. Increasing the inoculum ratio within the tested range resulted in higher Tartary buckwheat peptide yields, likely due to faster initial growth and increased protease secretion, which promoted the conversion of large proteins into small peptides. However, as the inoculum ratio increased further, the medium became nutrient-deficient, inhibiting the strain’s growth and enzyme production, thus reducing peptide content.

#### 3.2.4. Fermentation Temperature’s Effect on the Peptide Content in Fermentation Mash

At 30 °C, the peptide concentration reached (14.45 ± 0.41) mg/mL, significantly higher than at other fermentation temperatures (*p* < 0.05), as shown in Figure 2d. Fermentation temperature is critical for strain growth, as it influences the strains’ ability to produce enzymes. Based on these results, 30 °C was identified as the optimal fermentation temperature.

#### 3.2.5. Fermentation Time’s Effect on the Peptide Content in Fermentation Mash

Figure 2e shows that the highest peptide content, (13.2 ± 0.21) mg/mL, was obtained at 48 h of fermentation. Extending the fermentation time beyond this point likely caused *Lactiplantibacillus plantarum* to produce more proteases, leading to further hydrolysis of peptides into amino acids and thus a decrease in the intermediate peptide content.

#### 3.2.6. The Addition of Potassium Dihydrogen Phosphate’s Effect on the Peptide Content in Fermentation Mash

As shown in Figure 2f, increasing the content of KH_2_PO_4_ from 0.1% to 0.6% led to a gradual rise in peptide content, followed by a decline and stabilization, with no significant overall fluctuation. Peptide content ranged from 12.55 to 13.10 mg/mL when KH_2_PO_4_ addition was between 0.2% and 0.4%, which resulted in higher yields compared to other concentrations. Inorganic salts can significantly impact osmotic pressure in the medium, affecting microorganisms’ water absorption or loss, and thereby influencing their essential activities and fermentation capacity. Therefore, the ideal KH_2_PO_4_ addition was determined to be 0.3%.

### 3.3. Response Surface Optimization Results of Buckwheat Peptide Fermentation Conditions

#### 3.3.1. Response Surface Experimental Design and Results

To determine the optimal fermentation conditions for buckwheat peptides, moisture content (*A*), glucose addition (*B*), fermentation temperature (*C*), and inoculum ratio (*D*) were optimized using peptide content as the response variable. Response surface regression analysis was performed using Design-Expert 10 software with a Box-Behnken design. According to this matrix, 29 extractions were carried out to optimize this process. The design matrix was generated in software and the order of extractions was randomized to minimize the effects of unexpected variability on the system response. The experimental design and results are shown in Table 2.

#### 3.3.2. Regression Equation and Parameter Analysis

Quadratic multiple regression was applied to the data using Design-Expert 10 software, yielding the quadratic regression equations for the peptide content of each factor.

(2) $$\begin{array}{c} Y=16.84+1.38A-0.49B+2.64C+1.15D+0.16AB-0.40AC-0.53AD\\ +0.83BC-0.43BD+1.13CD-5.93A^{2}-1.17B^{2}\\ -1.28C^{2}-1.49D^{2} \end{array}$$

As seen in Table 3, the model had a *p* value < 0.0001, indicating a highly significant model. The lack of fit term had a *p* value of 0.0698 > 0.05, which is insignificant, demonstrating that the predicted values from the regression model aligned well with the measured values. The regression coefficient R^2^ of the model was 97.97%, and the adjusted R^2^Adj was 95.94%, indicating a strong fit between the model and the actual results, with the error falling within an acceptable range. This suggests that the fitted model provided a reliable mathematical description of the response results.

In the ANOVA, the primary terms *A*, *C*, and *D*, the interaction term *CD*, and the quadratic terms *A*^2^, *B*^2^, *C*^2^, and *D*^2^ had a highly significant effect (*p* < 0.01). The interaction term BC showed a significant effect (*p* < 0.05), while the rest were not significant. Based on the *F* values, the impact of each factor on the peptide content in the fermentation supernatant was ranked as fermentation temperature (*C*) > moisture content (*A*) > inoculum ratio (*D*) > glucose addition (*B*).

#### 3.3.3. Response Surface Optimization and Analysis

**Figure 3:**
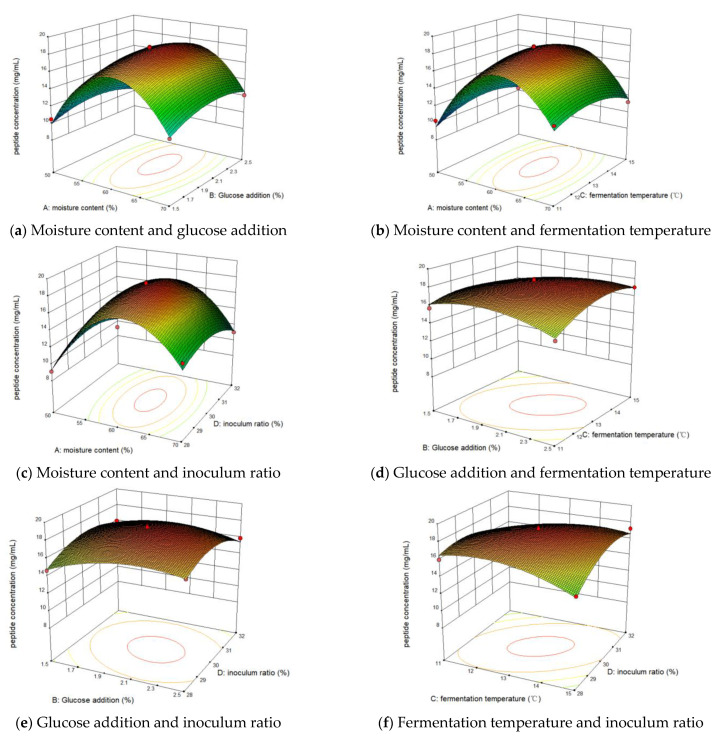
Effect of four-factor interaction on polypeptide content.

Figure 3’s surfaces and contours illustrate how two interrelated parameters influenced the polypeptide yield. The tighter the contours and the steeper the surfaces, the more susceptible these two parameters were to the effects of polypeptide yield. The change in color from blue to red represents the response value from low to high. The contour plots of the interaction between glucose addition and fermentation temperature (Figure 3d), as well as fermentation temperature and inoculum ratio, are elliptical, indicating a significant combined effect on peptide content (Figure 3f). This aligns with the ANOVA data analysis. The contour plots of the other factors are circular, suggesting that their interactions were not significant (Figure 3a–c,e). Figure 3d,f show that with fixed moisture content, inoculum, and glucose addition, the peptide content changed significantly with temperature. The content first increased slowly, then decreased, indicating that temperature significantly affected microbial growth and enzyme production. The results suggest that peptide yield is more sensitive to changes in incubation temperature than to changes in inoculum and glucose addition.

#### 3.3.4. Determination of Optimal Values and Validation Experiments of the Regression Model

The regression model predicted that the highest peptide content of 21.62 mg/mL could be achieved under the following conditions: 60.87% moisture content, 12.87% inoculum ratio, 2.08% glucose addition, and a fermentation temperature of 30 °C. A confirmatory test was conducted using these predicted values, with slight adjustments based on practical considerations. The final process conditions were set at 60% moisture content, 12.87% inoculum ratio, and 2% glucose addition. The experimental peptide content obtained was (22.18 ± 1.02) mg/mL, closely matching the predicted value of 21.62 mg/mL, demonstrating that fermentation with *Lactiplantibacillus plantarum* increased peptide yield by 2.59%.

## 4. Discussion

Based on experimental data from the fermentation of Tartary buckwheat, the optimal culture time for *Lactiplantibacillus plantarum* was found to be 48 h (Figure 2e). During this time, the strain entered the late logarithmic phase (according to pre-test data), and the protease released by the bacterial cells hydrolyzed proteins into peptides. Extending the incubation beyond this phase can result in insufficient biomass, and longer fermentation times may lead to the degradation of bacterial cells. By analyzing the fitted model derived from the response surface methodology (RSM), the optimal fermentation conditions were predicted to be 60.87% moisture content, 12.87% inoculum ratio, 2.08% glucose addition, and a fermentation temperature of 30 °C. Considering both theoretical and practical aspects, the final fermentation parameters were adjusted to 60% moisture content, 12.87% inoculum ratio, 2% glucose addition, and 30 °C fermentation temperature. The experimentally obtained peptide content of (22.18 ± 1.02) mg/mL closely aligned with the predicted value of 21.62 mg/mL, further validating that fermentation with *Lactiplantibacillus plantarum* increases peptide production in buckwheat SSF products. Moreover, it was observed that solid-state fermentation significantly increased the activity of key hydrolases in Tartary buckwheat, such as β-glucosidase, CMCase, xylanase, and FPase. These enzymes contribute to the breakdown of cellulose and hemicellulose in Tartary buckwheat, releasing more bioactive compounds [22].

Single-factor tests and RSM analysis were used to optimize the strain type and fermentation parameters (moisture content, glucose addition, fermentation temperature, and inoculum ratio) to maximize peptide yield in Tartary buckwheat. For strain selection, the experiment showed that *Lactiplantibacillus plantarum* alone produced the best results. This differs from Zhang’s study [23], where the ratio of *S. cerevisiae* to *Lactiplantibacillus plantarum* was optimized for SSF, along with the strain addition ratio. Using *Lactiplantibacillus plantarum* B1-6, Xin Rui [24] optimized SSF conditions for soybean, identifying three optimal conditions. Their response value measured *Lactobacillus* production, which was slightly different from our experimental setup. Additionally, several strains have been used for Tartary buckwheat fermentation, including *Eurotium cristatum* YL-1 [25], *Rhizopus oligosporus* ATCC 64063 [26], and *Lactiplantibacillus plantarum* KT [27]. However, most studies focused on antioxidant activity or peptide purification and identification post-fermentation, with limited attention given to the peptide yield of buckwheat. Yu [28] conducted peanut polypeptide fermentation under the following conditions: 20 h cell age, 3.0 mL bacterial suspension volume, 40 °C fermentation temperature, 15 mL nutrient salt solution, 42 h fermentation time, and a 45 °C water bath for 3 h. These conditions offer new insights, as certain experimental factors remain unexplored. Furthermore, Wu et al. [29] adjusted the SSF of walnut protein meal (WPM) using *Bacillus subtilis* to achieve the highest degree of hydrolysis (DH), with optimal conditions identified as an 82.01 h fermentation period, 10.40 inoculum ratio, and 1.50 mL/g water concentration, resulting in a 41.80% DH. In summary, fermentation variables such as cell age, bacterial suspension volume, and nutrient salt solution warrant further investigation.

The variance analysis indicated that fermentation temperature is the most critical factor in the treatment with *Lactiplantibacillus plantarum* (Table 3). Apart from 30 °C, most of the fermentation temperatures tested in this study had a positive effect on the strain’s growth and peptide production. This may be due to the significant influence of temperature and heat transfer on the growth of *Lactiplantibacillus plantarum* and the production of secondary metabolites during SSF. During SSF, a considerable amount of heat is generated, proportional to the microorganism’s metabolic activity. Heat exchange within the SSF system is closely tied to microbial metabolism and aeration of the fermentation system [30].

The peptide content in Tartary buckwheat was influenced by the fermentation conditions of *Lactiplantibacillus plantarum*, with moisture content being the second most important factor. Previous studies have shown that excessively high or low moisture levels can inhibit normal bacterial growth [31]. Our results confirm earlier findings that *Lactiplantibacillus plantarum* produces more peptides at intermediate moisture levels during SSF [32]. Higher inoculum ratio within the tested range led to greater activity, possibly due to faster initial growth rates. Meanwhile, glucose addition had a minimal effect on peptide content.

The bioactive components in Tartary buckwheat have been found to regulate metabolic diseases [33]. Several natural peptides derived from buckwheat seeds exhibit multifunctional properties, such as antimicrobial peptides, trypsin inhibitors, antitumor proteins, antihypertensive peptides, and antioxidant peptides. The antitumor and trypsin inhibition activities are linked to the specific active sites within the peptide molecules, while lipid-lowering and blood pressure-reducing effects are likely related to the unique amino acid composition of buckwheat protein, as hydrolyzed small peptides retain these bioactivities. Buckwheat peptides hold great potential in the fields of functional food and traditional medicine [34].

## 5. Conclusions

The results of this study demonstrated that the peptide yield of Tartary buckwheat SSF products can be significantly increased (*p* < 0.05) using *Lactiplantibacillus plantarum* ATCC 14917. Under optimal fermentation conditions (60% moisture content, 12.87% inoculum ratio, 2% glucose addition, and a fermentation temperature of 30 °C), the peptide content of SSF products reached (22.18 ± 1.02) mg/mL, representing a 2.59% increase compared to the theoretical value (*p* < 0.05).

Tartary buckwheat protein typically suffers from low digestibility, but Tartary buckwheat polypeptides can be directly absorbed by the human intestine due to their small molecular size and rapid absorption. *Lactiplantibacillus plantarum*, as a probiotic, improves the safety of Tartary buckwheat polypeptides prepared through microbial fermentation. Converting Tartary buckwheat protein into polypeptides maximizes the use of Tartary buckwheat resources, significantly increases the added value of its products, and expands the market applications and economic potential of Tartary buckwheat polypeptides in the food industry.

## Figures and Tables

**Table 1:** Codes and levels of independent variables used for response surface analysis.

Factor	Level		
	−1	0	1
*A* moisture content (%)	50	60	70
*B* glucose addition (%)	1.5	2	2.5
*C* inoculum ratio (%)	11	13	15
*D* fermentation temperature (°C)	28	30	32

**Table 2:** Response surface experiment design and results.

Run	Factors				Response
	*A* Moisture Content (%)	*B* Glucose Addition (%)	*C* Fermentation Temperature (°C)	*D* Inoculum Ratio (%)	*Y* Peptide Concentration (mg/mL)
1	0	0	0	0	18.43
2	1	0	1	0	11.23
3	−1	−1	0	0	10.49
4	0	0	−1	−1	15.99
5	0	0	1	1	17.22
6	0	1	−1	0	14.95
7	1	0	0	1	11.03
8	0	0	0	0	17.68
9	0	1	0	1	15.92
10	1	−1	0	0	11.43
11	0	−1	0	−1	14.67
12	0	−1	0	1	15.99
13	−1	1	0	0	10.53
14	0	0	0	0	18.15
15	−1	0	1	0	10.33
16	0	0	0	0	18.33
17	−1	0	0	−1	9.09
18	0	0	0	0	18.43
19	−1	0	0	1	9.17
20	0	1	0	−1	16.30
21	0	−1	−1	0	15.73
22	0	1	1	0	17.04
23	0	0	1	−1	14.53
24	−1	0	−1	0	10.29
25	1	1	0	0	12.12
26	1	0	0	−1	13.05
27	1	0	−1	0	12.79
28	0	−1	1	0	14.50
29	0	0	−1	1	14.15

**Table 3:** Regression equation analysis of variance.

Source	Sum of Squares	df	Mean Square	*F* Value	*p*-Value Prob > *F*	Significance
Model	252.53	14	18.04	48.23	<0.0001	**
*A* moisture content (%)	5.71	1	5.71	15.26	0.0016	**
*B* Glucose addition (%)	0.73	1	0.73	1.95	0.1848	
*C* fermentation temperature (°C)	9.96	1	9.96	26.64	0.0001	**
*D* inoculum ratio (%)	3.93	1	3.93	10.52	0.0059	**
*AB*	0.11	1	0.11	0.28	0.6034	
*AC*	0.64	1	0.64	1.71	0.2119	
*AD*	1.1	1	1.1	2.95	0.1080	
*BC*	2.76	1	2.76	7.37	0.0168	*
*BD*	0.72	1	0.72	1.93	0.1863	
*CD*	5.13	1	5.13	13.72	0.0024	**
*A^2^*	228.15	1	228.15	610.04	<0.0001	**
*B^2^*	8.81	1	8.81	23.57	0.0003	**
*C^2^*	10.64	1	10.64	28.45	0.0001	**
*D^2^*	14.32	1	14.32	38.29	<0.0001	**
Residual	5.24	14	0.37			
Lack of Fit	4.84	10	0.48	4.9	0.0698	Not significance
Pure Error	0.4	4	0.099			
Cor Total	257.76	28				
	R^2^	0.9797		R^2^_Adj_	0.9594	

Note: The model was statistically significant (*p* < 0.05). * *p* < 0.05, significant difference; ** *p* < 0.01, extremely significant difference.

## Data Availability

The original contributions presented in the study are included in the article; further inquiries can be directed to the corresponding author.
